# Supplementary material for: Effective purifying selection in ancient asexual oribatid mites
Source: Nat Commun. 2017 Oct 12;8:873. doi: 10.1038/s41467-017-01002-8 (PMC5638860; doi:10.1038/s41467-017-01002-8)
Supplement: Supplementary file 1 — Supplementary Information [file 41467_2017_1002_MOESM1_ESM.pdf]

Supplementary Table 1: NCBI accession numbers of partial coding sequences of *ef1α* and *hsp82* and partial sequences of 18S rDNA of 30 oribatid mite species generated by Domes *et al*. [1]. Sequences were used for construction of a phylogenetic tree (Fig. 1) and branch-specific dN/dS ratio analyses. Because sequences of *Nothrus palustris* were not part of the study of Domes *et al*. [1] and not available from NCBI, they were extracted from the transcriptome data. Order of species reflects the arrangement of species in the phylogenetic tree from top to bottom.

**Species**	**Reproductive mode**	**Accession number**		
		ef1α	***hsp82***	**18S rDNA**
Tectocepheus velatus	asexual	EF093763	EF093770	EF093781
Eupelops plicatus	sexual	AY632797	DQ090783	EF091419
Achipteria coleoptrata	sexual	AY632776	EF081335	EF091418
Eutegaeus curviseta	sexual	EF081326	DQ090789	EF081297
*Carabodes femoralis**	sexual	EF081325	DQ090786	EF091429
Nothrus silvestris bistilus	asexual	EF081323	EF081333	EF081305
Nothrus truncatus	asexual	EF081322	EF081334	EF081306
Nothrus palustris	asexual	-	-	-
Novonothrus flagellatus	sexual	EF081324	DQ090801	EF081307
Camisia biurus	asexual	EF081312	EF081331	EF081302
Camisia spinifer	asexual	EF081313	EF081332	EF091420
Crotonia brachyrostrum	sexual	EF081314	DQ090796	EF081303
Crotonia cf caudata	sexual	EF081315	DQ090795	EF081304
Platynothrus peltifer	asexual	AY632851	DQ090793	EF091422
Heminothrus paolianus	asexual	EF081316	DQ090794	EF091423
Nanhermannia coronata	asexual	AY632825	DQ090799	EF091421
Archegozetes longisetosus	asexual	EF081321	DQ090798	AF022027
Trypochthonius americanus	asexual	EF081317	EF081337	EF081298
Trypochthoniellus crassus	asexual	EF081320	EF081336	EF081300
Mainothrus badius	asexual	EF081318	EF081338	EF081301
Mucronothrus nasalis	asexual	EF081319	DQ090797	EF081299
Malaconothrus gracilis	asexual	EF081311	EF081339	EF091424
Hermannia gibba	sexual	EF081327	DQ090800	EF091426
Steganacarus magnus	sexual	AY632837	DQ090781	AF022040
Atropacarus striculus	asexual	EF081309	DQ090782	EF091416
Rhysotritia duplicata	asexual	EF081310	DQ090780	EF091417
Nehypochthonius porosus	asexual	EF081328	DQ090779	EF081308

Lohmannia banksi	asexual	EF081330	DQ090777	AF022036
Hypochthonius rufulus	asexual	AY632861	DQ090776	EF091427
Eniochthonius minutissimus	asexual	EF081329	DQ090773	EF091428

* *Carabodes subarcticus* for 18S rDNA

Supplementary Table 2: Details on oribatid mite sampling and transcriptome sequencing.

Species	Reproduc-tive mode	Sampling locality	No of pooled individuals	Sequencing provider	Sequencing platform
Achipteria coleoptrata	sexual	Solling	20	GATC Biotech	HiSeq 2500 125 bp PE
Hermannia gibba	sexual	Göttinger Wald	10	GATC Biotech	HiSeq 2500 125 bp PE
Hypochthonius rufulus	asexual	Göttinger Wald	50	GATC Biotech	HiSeq 2500 125 bp PE
Nothrus palustris	asexual	Göttinger Wald	3	GATC Biotech	HiSeq 2500 125 bp PE
Platynothrus peltifer	asexual	Solling	1	TAL	MiSeq 250 bp PE
Steganacarus magnus	sexual	Göttinger Wald	1	TAL	MiSeq 250 bp PE

Supplementary Table 3: Overview of the assembly and filtering processes. After assembly, transcripts were cleaned and then filtered for the most abundant isoform (i.e. most abundant allele in the population) using an RPKM based approach. The filtered transcript set was assessed for quality using BUSCO ('complete' are core arthropod genes assembled to full length). For subsequent analyses of purifying selection, ORFs were predicted.

**Species**	**No of raw reads [M]**	**Contig N50 [bp]**	**No of transcripts**	**No of filtered transcripts**	**No of ORFs**	**BUSCO complete [%]**	**BUSCO fragments [%]**	**BUSCO missing [%]**
Achipteria coleoptrata	f: 67.03<br>r: 70.30	1,318	123,336	34,262	16,070	93.20	1.80	5.00
Hermannia gibba	f: 65.45<br>r: 68.47	1,481	142,723	39,974	19,108	95.70	0.50	3.80
Hypochthonius rufulus	f: 54.42<br>r: 60.23	1,204	59,116	35,595	15,507	90.10	5.40	4.50
Nothrus palustris	f: 68.44<br>r: 71.76	1,703	118,984	30,608	16,397	95.00	0.80	4.20
Platynothrus peltifer	f: 8.97<br>r: 11.35	702	85,238	68,779	24,108	83.40	11.70	4.90
Steganacarus magnus	f: 13.42<br>r: 16.83	1,761	73,855	35,286	21,131	92.60	2.30	5.10

Supplementary References

1. Domes, K., Norton, R. A., Maraun, M. & Scheu, S. Reevolution of sexuality breaks Dollo's law. *Proc. Natl. Acad. Sci. U. S. A.* **104,** 7139–7144 (2007).
