# Supplementary material for: Effective purifying selection in ancient asexual oribatid mites
Source: Nat Commun. 2017 Oct 12;8:873. doi: 10.1038/s41467-017-01002-8 (PMC5638860; doi:10.1038/s41467-017-01002-8)
Supplement: Supplementary file 3 — Description of Additional Supplementary Files [file 41467_2017_1002_MOESM3_ESM.pdf]

File Name: Supplementary Data 1
Description: Overrepresented Gene Ontology terms in orthologs with strong selection in asexual oribatid mites. The table contains Gene Ontology terms that were overrepresented in the set of 67 orthologs that yielded significantly lower dN/dS ratios at asexual as compared to sexual branches, results of Fisher´s exact tests and names of test and reference sequences (see Methods).
